# Growth and metabolic characteristics of oleaginous microalgal isolates from Nilgiri biosphere Reserve of India

**DOI:** 10.1186/s12866-017-1144-x

**Published:** 2018-01-03

**Authors:** Kalaiselvi Thangavel, Preethi Radha Krishnan, Srimeena Nagaiah, Senthil Kuppusamy, Senthil Chinnasamy, Jude Sudhagar Rajadorai, Gopal Nellaiappan Olaganathan, Balachandar Dananjeyan

**Affiliations:** 1 Department of Agricultural Microbiology, Tamil Nadu Agricultural University, Coimbatore, 641003 Tamil Nadu India; 2 Biotechnology Division, Aban Infrastructure Pvt Ltd, Janpriya Crest, 113, Pantheon Road, Egmore, Chennai, 600 008 India; 3 Forest College & Research Institute, Tamil Nadu Agricultural University, Mettupalayam, 641301 Tamil Nadu India

**Keywords:** Biodiesel, *Acutodesmus*, *Chlorella*, *Chlamydomonadales*, *Hindakia*, Nilgiri biosphere

## Abstract

**Background:**

Renewable energy for sustainable development is a subject of a worldwide debate since continuous utilization of non-renewable energy sources has a drastic impact on the environment and economy; a search for alternative energy resources is indispensable. Microalgae are promising and potential alternate energy resources for biodiesel production. Thus, our efforts were focused on surveying the natural diversity of microalgae for the production of biodiesel. The present study aimed at identification, isolation, and characterization of oleaginous microalgae from shola forests of Nilgiri Biosphere Reserve (NBR), the biodiversity hot spot of India, where the microalgal diversity has not yet been systematically investigated.

**Results:**

Overall the higher biomass yield, higher lipid accumulation and thermotolerance observed in the isolated microalgal strains have been found to be the desirable traits for the efficient biodiesel production. Species composition and diversity analysis yielded ten potential microalgal isolates belonging to Chlorophyceae and Cyanophyceae classes. The chlorophytes exhibited higher growth rate, maximum biomass yield, and higher lipid accumulation than Cyanophyceae. Among the chlorophytes, the best performing strains were identified and represented by *Acutodesmus dissociatus* (TGA1), *Chlorella* sp. (TGA2), *Chlamydomonadales* sp. (TGA3) and *Hindakia tetrachotoma* (PGA1). The *Chlamydomonadales* sp. recorded with the highest growth rate, lipid accumulation and biomass yield of 0.28 ± 0.03 day^−1^ (μ_exp_), 29.7 ± 0.69% and 134.17 ± 16.87 mg L^−1^ day^−1^, respectively. It was also found to grow well at various temperatures, *viz.*, 25 °C, 35 °C, and 45 °C, indicating its suitability for open pond cultivation. The fatty acid methyl ester (FAME) analysis of stationary phase cultures of selected four algal strains by tandem mass spectrograph showed C16:0, C18:1 and C18:3 as dominant fatty acids suitable for biodiesel production. All the three strains except for *Hindakia tetrachotoma* (PGA1) recorded higher carbohydrate content and were considered as potential feed stocks for biodiesel production through hydrothermal liquefaction technology (HTL).

**Conclusions:**

In conclusion, the present investigation is a first systematic study on the microalgal diversity of soil and water samples from selected sites of NBR. The study resulted in isolation and characterization of ten potent oleaginous microalgae and found four cultures as promising feed stocks for biodiesel production. Of the four microalgae, *Chlamydomonadales* sp. (TGA3) was found to be significantly thermo-tolerant and can be considered as promising feedstock for biodiesel production.

**Electronic supplementary material:**

The online version of this article (10.1186/s12866-017-1144-x) contains supplementary material, which is available to authorized users.

## Background

The total global fossil fuel reserves reached 1700 billion barrels of oil and 187.1 trillion cubic meters of natural gas at the end of 2014 (Oil and Gas Journal, 2015). At current rates of consumption of 4211.1 million tonnes of oil per year and 3065.5 billion cubic meters of natural gas per year, the existing oil and natural gas reserves would be exhausted in another 52.2 years and 54.1 years respectively (Oil and Gas Journal, 2015).

As the non-renewable energy resources are going to exhaust and energy demand is rising, the energy transition to renewable resources is inevitable. Biomass and biofuels have an important role to play in this transition. The first generation potential biofuel feed stocks are edible oils derived from soybean, rapeseed, coconut, and palm; however, there is a competition of usage between food versus fuel. The second generation biofuels such as ethanol produced from lignocellulosic biomass have still not significantly developed due to expensive pretreatment procedures. Furthermore, biodiesel production from non-edible oil resources such as Jatropha failed to meet the growing energy needs because of poor yield and extensive labor cost incurred during harvesting. In this context, microalgae have emerged as potential cell factories for the efficient production of biodiesel.

The identification and characterization of suitable algal strains with high lipid productivity particularly of neutral lipids and ability to grow in diverse environmental conditions are essentially required for the efficient microalgal biofuel production [1]. Over the past decades, several studies including the Aquatic Species Program (ASP) have investigated the selection of lipid-rich microalgae [2]; however, only a few strains have gone beyond the laboratory. Although the highest microalgal oil content reported so far is 80% by dry weight, most of the commercially used algae have only oil contents between 20% and 50% [3]. Therefore, the identification and characterization of potential microalgae found in a wide range of natural habitats for the maximum production of biodiesel using improved cultivation practices and by the manipulation of the genetic system of the selected cultures are highly desirable.

Microalgae are typically found as a mixed consortium and the population dynamics of the microalgae in any habitat is complex [4]. The ability of algae to survive and proliferate in diverse environmental conditions, to a large extent, reflects in their tremendous diversity and an ability to modify lipid metabolism efficiently in response to the changes in environmental conditions [1]. Certainly, the same strain of microalgae species could exhibit different physiological activities and metabolite production in response to the habitat and physico-chemical conditions. Strains belonging to the same species or closely related species, but isolated from different environments, might behave differently and produce different biomolecules [5]. Therefore, the phenotypic diversity is of a greater importance than genotypic diversity, which further enhances the opportunity to obtain newer or better isolates from different environments. The diversity is even higher in an immaculate ecosystem. The present study thus aimed at identification, isolation, and characterization of novel microalgae strains from the unexplored forest ecosystem, the Nilgiri Biosphere Reserve (NBR).

**Fig. 1:**
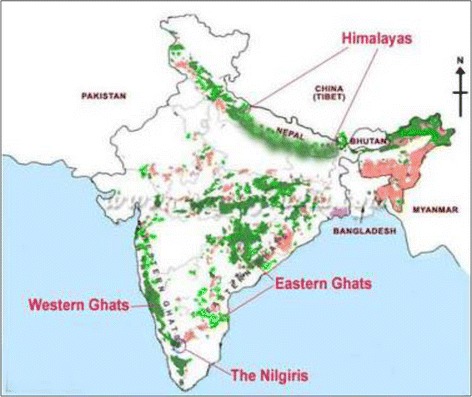
Location map showing the sampling site, the Western ghats of Nilgiri Biosphere Reserve (NBR), India

The NBR in the Western Ghats (Fig. 1) is one of the mega biodiversity hotspots of the world and is also rich in microorganisms. The shola forests in this reserve are a special ecological niche owing to their unique micro and macroclimatic conditions. The fertile soils with high moisture holding capacity of these forests offer excellent conditions for the proliferation of diverse microorganisms including microalgae [6].

The ability of microalgae to produce biodiesel depends on lipids, which are contained in the microalgae. Intracellular neutral lipids can be detected by Nile red staining under a fluorescence microscope. Besides lipid productivity, the fatty acid composition is also an important factor in screening microalgal strains for biodiesel production. Thus, biodiesel properties of microalgal oils depend essentially on their fatty acid profile. Most of the fresh water algae produce fatty acids of C14:0, C16:0, C18:1, C18:2 and C18:3 in larger quantity and the relative proportions of other fatty acids are highly dependent on species [7]. Thus, our efforts focused on surveying the natural diversity of microalgal strains for the production-related properties. The study further evaluated the growth kinetics, macromolecular constituents, and fatty acid profile for their suitability as a biodiesel feedstock.

The present study extensively investigated the growth and metabolic characterization of identified four green microalgae, *viz*., *Acutodesmus dissociatus, Chlorella* sp.*, Chlamydomonadales* sp., and *Hindakia tetrachotoma* with high lipid contents and biomass yield at the studied growth phase. *Chlamydomonadales* sp. can grow even at 45 °C thus showing maximum thermal tolerance to be a potential biodiesel feedstock for open pond cultivation with low risk of contamination.

## Results and discussion

The samples were collected from NBR’s natural and well managed TNAU and Sethumadai ecosystems and the collected samples were enriched in BG11 and Chu13 media for 40 days and the microalgal species composition was studied.

### Microalgal species composition

The sampling sites represent both terrestrial and aquatic environments of shallow and temporal water bodies. The strains from aquatic environments are expected to be potential feed stocks for open raceway cultivation. Microclimate in these sampling sites frequently varies from optimal growth condition to unfavourable situation (low and high temperature, low and high light intensity, low and high rainfall and dry, hot or cold weather). Thus sampling in these locations was considered advantageous, since the microalgae exposed to unfavorable conditions could accumulate more photosynthates as starch or lipid to tide over the unfavorable conditions.

The enrichment culture studies showed microalgal community-wise succession, which was initially dominated by unicellular green algae and followed by diatoms. The diatoms were isolated within their growth period of 20 days, beyond which, they subsided. There was a subsequent shift to dominance mainly by filamentous green algae. Many algal strains could not survive till the end of enrichment, which might be due to changing nutrients and growth conditions. The microalgal strains that were stable, grew faster and surpassed the succession were reserved for further investigation.

The microscopic observation of the enriched water and soil samples indicated the presence of a community of algal strains including unicellular, filamentous green and blue green algae, heterocystous and non-heterocystous blue green algae, and diatoms. In general, among the different forms, unicellular blue green and green algae dominated the filamentous forms (Additional file 1: Table S1). This could be attributed to a wider adaptation of unicellular soil microalgae to rapidly changing and adverse environmental conditions. In addition, their resting cysts might easily survive transportation over long distances in the air and impart resistance to drought, high and low light intensities, and high UV radiation [8]. A high abundance of unicellular blue green algae *Pleurocapsa, Gloeocapsa,* and *Phormidium* in littoral zones and some filamentous algae (*Nostoc*, *Scytonema*) in water lines have also been reported earlier [9]. The heterocystous blue green algae, *Tolypothrix* and *Calothrix,* are typical components of a sub-surface littoral community. Since the soil samples analyzed in this study were collected from the surface area, they might harbor more unicellular algae than filamentous forms. Interestingly, the co-existence of heterocystous and non-heterocystous blue green algae was noticed in all the samples examined irrespective of the sampling sites. This observation is contrary to the findings of Whitton and Potts [10], who reported that non-heterocystous cyanobacteria are more abundant than heterocystous algae in diverse ecological environments. Light conditions and temperature directly influence the growth of green and blue green algae (duration and intensity), thus temperature change in the environment at the time of the sampling was also recorded and both green and blue green algae were observed to be particularly sensitive to high light intensities. At high light intensities, the photosynthetic capacity decreases and the growth of blue-green and green algae is inhibited [11]. In the present study, an equal proportion of unicellular blue green and green algal forms were observed in the samples drawn during spring. Favorable climatic conditions, such as moderate temperature and low light intensities, were prevalent at the study sites during the sampling period and might have favored the growth of both the algal forms.

Among soil properties, pH is an important factor affecting growth, establishment, and diversity of cyanobacteria. A pH value, from neutral to slightly alkaline, has been reported for the optimum growth of cyanobacteria [12]; the same was further confirmed in our analysis. The dominance of green algae at the study sites could be attributed to the acidic nature of the samples, favoring a higher growth of green algae than blue green algae. Acidic soils are usually dominated by diatoms and green algae and normally lack blue-green algae [13]. Furthermore, it was also reported that the variations in the physico-chemical properties of the soil including pH, organic carbon, and nitrogen have lesser effects on green algae than other microalgae [14].

The present study also revealed the presence and dominance of Cyanophyceae in cultivable lands and the presence of Cyanophyceae, Chlorophyceae, and Bacillariophyceae in the forest ecosystem. The reports from the temperate forests and grasslands show that alkaline and nutrient rich soils favor a high group-diversity comprising of cyanobacteria, diatoms, xanthophytes, and green algae, whereas acidic and nutrient poor soils are often inhabited by green algae and diatoms [15]. Cyanobacteria are reported to prefer slightly higher temperature (32–37°C) over chlorophytes (25°C) [16]; this could be responsible for the lesser number of cyanobacteria observed in the forest ecosystem.

### Microscopic characterization and identification of microalgae

Microalgal strains were tentatively identified using the keys given in the monographs [17, 18]. The morphological comparison of isolated cultures with other described microalgae suggested that these strains belong to different genera as presented in (Additional file 1: Table S1). Shola forest soils were found to be enriched with filamentous heterocystous blue green algal strains, *Anabaena, Nostoc, Tolypothrix, Calothrix, Cylindrospermum,* and *Gloeotrichia;* while *Tolypothrix* was the only algal strain found in water samples of forest ecosystem and cultivable lands. As reported earlier [9], *Tolypothrix* dominates the wet land ecosystem. Filamentous nonheterocystous forms were represented by *Oscillatoria, Spirulina,* and *Lyngbya;* while *Aphanocapsa, Microcystis, Gloeocapsa, Chroococcus, Synechocystis, Synechococcus,* and *Merismopodia* were the unicellular blue green algal forms.

Unicellular green algal strains, *Chlorella, Acutodesmus, Chlorococcum, Chlamydomonadales, Dactylococcus, Hindakia, Botryococcus,* and *Trentepohlia* were also recorded in all types of ecosystems. *Spirogyra* was found in the water sample obtained from a cultivable ecosystem rich in nutrients. Some of the enriched water sources contained mostly diatoms of *Navicula* sp. Unicellular chlorophyceae alga *Trentepohlia* was also obtained from bark samples containing lichen.

### Diversity analyses

The microalgal diversity among the soil and water samples collected from the forest and agricultural ecosystems was estimated by α-diversity indices (Table 1). The species richness assessed by the Shannon Weiner index (H′) ranged between 0 and 1.551. The values recorded for evenness (Shannon’s Equitability) and dominance (Simpson index) ranged from 0 to 1.0. For greater microalgal diversity, the value of the Shannon Wiener index and Shannon’s Equitability index should be high and the value of Simpson index should be low. The microalgae obtained from the soil samples of Parambikulam and Top Slip were recorded with the highest Shannon diversity index (H′ = 1.551 and 1.550, respectively) and thus represented highest species richness, harboring a diverse variety of unicellular and filamentous microalgae. The highest Shannon equitability index was observed in the soil samples of Mel Kuntha, Ooty (E_H_ = 1.0). Rose Garden, Ooty, and Pykara Dam soil samples registered maximum value (1.0) for the Simpson dominance index (Table 1). The comparison of the number and the form of algae (Additional file 1) revealed the richness of algae in soil samples of Parambikulam, Top slip, and TNAU, Coimbatore. The tropical montane evergreen forests (shola) are characterized by a closed canopy, low light penetration, continuous addition of litter fall, the accumulation of eluted nutrients, and high humidity [19]. These microclimatic conditions favored rich algal diversity. The closed and undisturbed shola forest soils harbored a number of different kinds of algae than the sites with anthropogenic activity. Thus, human interference was the major limiting factor that affects the species richness.

**Table 1:** Microalgal diversity analysis

Location	Type of sample	Diversity Indices		
		Species richness (Shannon – Weiner index H′)	Evenness (Shannon’s equitability E_H_)	Dominance (Simpson’s index)
Maize field of eastern block, TNAU	Soil	1.474	0.916	0.200
Wet land, TNAU	Water	1.477	0.824	0.267
Sethumadai, NBR	Soil	0.956	0.870	0.333
Top Slip, NBR	Soil	1.550	0.960	0.151
Top Slip, NBR	Water	1.168	0.843	0.361
Parambikulam, NBR	Soil	1.551	0.967	0.167
Parambikulam, NBR	Water	1.418	0.881	0.272
Kamarajar sagar dam, NBR	Water	1.332	0.960	0.100
Kamarajar sagar dam, NBR	Soil	0.636	0.918	0.333
Kamarajar sagar dam, NBR	Bark	0.000	0.000	0.000
Shola forest, NBR	Soil	1.242	0.896	0.200
Mel Kuntha, NBR	Soil	0.693	1.000	0.000
Rosegarden, Ooty	Soil	0.000	0.000	1.000
HPF,Ooty	Soil	1.242	0.896	0.200
Pykara, NBR	Water	0.637	0.918	0.330
Pykara, NBR	Soil	0.000	0.000	1.000

Due to low species diversity and almost an equal proportion of different algae, the highest dominance was evident for the algal samples of Rose garden and Pykara, Ooty. The diversity analyses indicated that Parambikulam and Top slip had the highest richness of microalgae and may be represented as the most promising sites for prospecting and evolving newer microalgal strains with higher lipid content and higher biomass yield.

### Culture identification and choice of growth medium

The majority of soil organisms particularly microalgae are difficult to culture under laboratory conditions. The prospecting sites yielded only ten stable isolates. These ten cultures including seven green algae and three blue-green algae were identified and represented by *Acutodesmus dissociatus* (TGA1) *Chlorella* sp. (TGA2), *Chlamydomonadales* sp. (TGA3), *Chlorella* sp. (TGA4), *Chlamydomonadales* sp. (TGA5), *Hindakia tetrachotoma* (PGA1) *Chlorella* sp. (PGA2), *Tolypothrix* sp. (PBGA1), *Tolypothrix* sp. (PBGA2) and *Oscillatoria* sp. (PBGA3). These isolates were grown in three different media including BG11, modified BBM, and Bristol. Modified BBM was found to greatly influence the growth of all the green algae, whereas blue green algal cultures exhibited maximum growth in BG11.

### Comparison of growth pattern of microalgal isolates

**Fig. 2:**
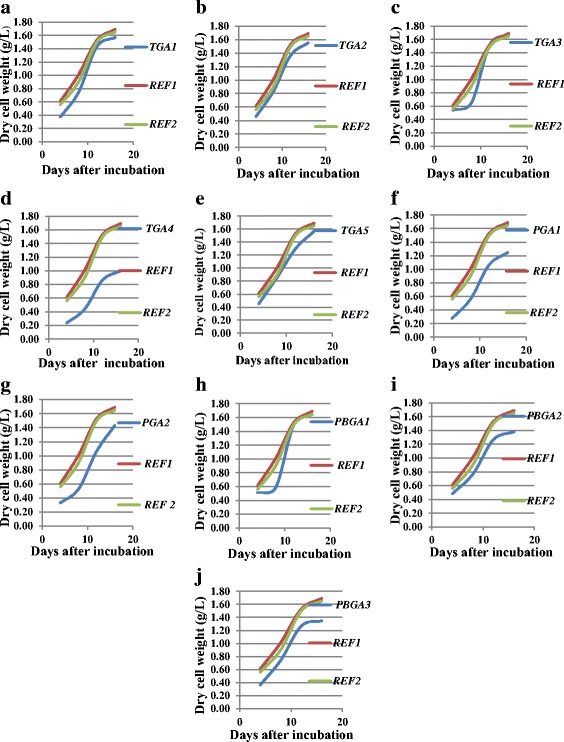
Comparison of growth pattern of ten microalgal cultures along with standard cultures, REF1-*Botryococcus* sp. and REF2-*Neochloris oleoabundans.* **a** Growth curve of TGA1-*Acutodesmus dissociatus,* **b** Growth curve of TGA2-*Chlorella* sp., **c** Growth curve of TGA3-*Chlamydomonadales* sp., **d** Growth curve of TGA4-*Chlorella* sp., **e** Growth curve of TGA5-*Chlamydomonadales* sp., **f** Growth curve of PGA1-*Hindakia tetrachotoma*, **g** Growth curve of PGA2-*Chlorella* sp., **h**. Growth curve of PBGA1-*Tolypothrix* sp., **i** Growth curve of PBGA2-*Tolypothrix* sp., **j** Growth curve of PBGA3-*Oscillatoria* sp. Data are given as means, *n* = 3

To determine and compare the growth pattern of microalgal cultures in terms of biomass yield, doubling time, and specific growth rate, all microalgal cultures were grown in batch culture up to 16 days at 24 ± 1 ^ο^C. Immediately after the incubation, an exponential growth was observed in most of the strains except TGA3 (*Chlamydomonadales* sp.) and PBGA1 (*Tolypothrix* sp.), where a prolonged lag phase was observed until the 8th day (Fig. 2). Typically, an inoculum from a healthy log phase culture exhibits very short lag phase [20], when transferred into a fresh medium under identical growth conditions. Further, the faster adaptability of the cultures to a new environment might be the reason for the absence of lag phase in most of the cultures under the study. The exponential growth occurred until the 12th day in all cultures except TGA5 and PGA2 (Table 2; Fig. 2). These two cultures continued to grow until the 16th day. The two strains, TGA3 and PBGA1, exhibited the most rapid growth, showed log phase between 8th and 12th day, recorded maximum dry biomass yield at 12th day and reached plateau thereafter. Overall, maximum growth occurred between the 8th and 12th day. Observations during the growth experiments also revealed that harvesting of the microalgal cultures would be easy since the cultures settled readily at the bottom under static culture. The average dry biomass yield of 16-day old cultures ranged from 1.00 g L^−1^ to 1.67 g L^−1^. The isolate TGA3 obtained from the soil samples of Top Slip (NBR) recorded a value of 1.67 g L^−1^, which was similar to the bench mark strain *Botryococcus* sp. (REF1). Except for TGA4, the other strains recorded with a high average dry biomass values.

**Table 2:** Growth characteristics of different microalgal isolates

Algal strain	Generation time (in days)	Dry cell weight (16 day old cultures) (g L^−1^)	Specific growth rate μ_exp_ (day ^−1^)	Total Chlorophyll (16 day old cultures) (mg L ^−1^)
*Acutodesmus dissociatus* TGA1	4.0 ± 0.12^cd^	1.57 ± 0.032^bc^	0.23 ± 0.05^bc^	6.7 ± 1.20^g^
*Chlorella* sp. TGA2	5.0 ± 0.17^b^	1.55 ± 0.036^b^	0.25 ± 0.03^a,b^	21.2 ± 0.73^a^
*Chlamydomonadales* sp. TGA3	3.8 ± 0.12^e^	1.67 ± 0.039^ab^	0.28 ± 0.03^a^	17.3 ± 1.25^bc^
*Chlorella* sp. TGA4	4.0 ± 0.20^cd^	1.00 ± 0.023^e^	0.10 ± 0.03^g^	19.0 ± 0.51^ab^
*Chlamydomonadales* sp. TGA5	6.0 ± 0.12^a^	1.56 ± 0.036^bc^	0.13 ± 0.03^f^	15.2 ± 0.82^cd^
*Hindakia tetrachotoma* PGA1	4.0 ± 0.12^cd^	1.25 ± 0.029^d^	0.18 ± 0.03^de^	10.4 ± 1.57^ef^
*Chlorella* sp. PGA2	4.9 ± 0.17^b^	1.43 ± 0.033^c^	0.12 ± 0.01^f^	16.5 ± 1.27^bcd^
*Tolypothrix* sp. PBGA1	1.0 ± 0.12^f^	1.64 ± 0.038^abc^	0.18 ± 0.03 ^de^	14.1 ± 0.69^cd^
*Tolypothrix* sp. PBGA2	5.2 ± 0.2^b^	1.38 ± 0.032^c^	0.19 ± 0.02^cd^	14.6 ± 0.12^cd^
*Oscillatoria* sp. PBGA3	4.4 ± 0.12^c^	1.35 ± 0.031^cd^	0.14 ± 0.04^ef^	8.4 ± 0.8 5^fg^
*Botryococcus* sp. REF1	5.2 ± 0.12^b^	1.69 ± 0.039^a^	0.23 ± 0.08^bc^	15.2 ± 0.30^cd^
*Neochloris oleoabundans* REF2	6.0 ± 0.12^a^	1.65 ± 0.038^abc^	0.24 ± 0.08^bc^	13.2 ± 1.22^de^

In a column, means followed by a common letter in superscript are not significantly different at 5%

The generation time of different cultures (Table 2) varied from one day (PBGA1) to a maximum of six days (TGA5). The highest biomass accumulation by the strain TGA3 demonstrated the most rapid growth with a doubling time of 3.8 ± 0.12 days, faster than REF1 (5.2 ± 0.12 days). The specific growth rate (μ_exp_) of the exponential phase cultures ranged from 0.28 to 0.10 μ_exp_ (day^−1^). The highest specific growth rate of 0.28 ± 0.03 μ_exp_ (day^−1^) was observed for TGA3, offering the lowest operating time required for single phase or biphasic mode of growth for biomass/biofuel production. Algal growth was also examined in terms of chlorophyll contents. A rapid growth of the strains TGA2, TGA4, and TGA3 ranging from 6.7 ± 1.20 mg L^−1^ (TGA1) to 21.2 ± 0.73 mg L^−1^(TGA2) was observed.

### Screening microalgae for neutral lipid production

**Fig. 3:**
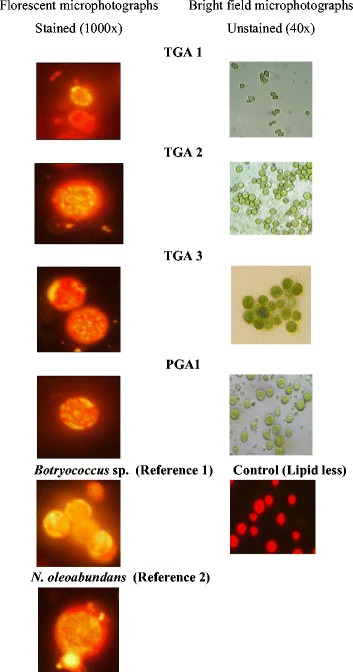
Nile red stained algal strains viewed at 1000× using a fluorescence microscope with 450–490 nm excitation and 570 nm emission filters (NIKON; Eclipse H600L). Neutral lipid globes in the cytosol were stained as yellow and chlorophyll autofluoresce as red

All strains were selected on the basis of lipid productivity by subjecting the isolates to Nile red staining. The intracellular lipid content of 12-day old oleaginous microalgal cultures from different environmental samples was qualitatively evaluated by the staining process. After staining with Nile red, strong fluorescence signals were detected. Among ten strains, intense yellow fluorescence was observed in four isolates, *viz*., TGA1, TGA2, TGA3, and PGA1 indicating their higher lipid accumulation (Fig. 3). An inherent problem with Nile red staining is its inability to distinguish between species having different cell wall structures because the intensity of staining also depends on cell wall permeability. Thus, the differences in lipid content obtained by Nile staining alone cannot be compared and hence microalgal cultures were further investigated gravimetrically for lipid content prior to the beginning of the stationary phase.

**Fig. 4:**
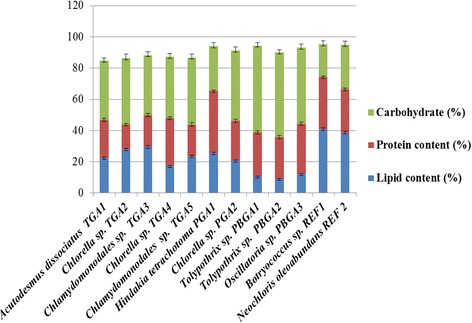
Comparison of macromolecular constituents of ten microalgal strains with standard cultures (REF1-*Botryococcus* sp. and REF2-*Neochloris oleoabundans*)*.* Data are given as means ± standard error, *n* = 3

The gravimetric analysis of lipid content of different isolates is illustrated in Fig. 4. The lipid accumulating capacity of the isolates varied largely indicating the significance of bioprospecting of algal species for biodiesel production (Fig. 4). Of the two algal families, the members of Chlorophyceae showed larger contents of total lipids than those reported for Cyanophyceae members. Besides, the possible differences in membrane permeability to Nile red, the variations in lipid content could also be attributed to the predominant reserve material such as starch in blue green algae and oil bodies in green algae [21].

It is noteworthy to mention that gravimetric lipid assay of all the green algae showed the accumulation of >20% of lipid. The total lipid accumulating capacity of the best performing strains after 16 days of incubation was in the order of TGA3 > TGA2 > PGA1 > TGA1. The presence of rigid cell wall in PGA1, TGA2, and TGA1 could be in part responsible for the low recovery of lipids from the cytosol. Neutral lipid content as determined by Nile red staining was found to be same as that determined gravimetrically.

It is quite interesting to note that microalgal species of the same genera showed varying levels of lipid accumulation. Among the different isolates of *Chlorella* sp., *Chlamydomonadales* sp*.*and *Tolypothrix* sp. a significant difference in lipid accumulation was observed. Such variation in lipid accumulation within the strains of a species from the same geographical region demonstrated that surveying microalgal isolates may not be sufficient, as different strains of the same species may have different lipid productivity [22]. Although the lipid content of microalgae remains constant when grown under identical growth conditions, every algal species exhibits a typical lipid profile. Therefore, the strain selection is critical for biodiesel production. The lipid fractions of the best performing strains were observed to contain commercially important volatile oils; this may improve their biomass value.

The study, thus revealed that the lipid content of 16 day old cultures of TGA1, TGA2, TGA3 and PGA1was significantly promising for biofuel production. The lipid content of these strains could further be enhanced by inducing physiological stress such as nutrient limitation, temperature, pH, or light stress. Furthermore, an inverse relationship between lipid and carbohydrate contents was noticed (Fig. 4). This could be due to sharing common precursors in the form of C3 metabolites, *viz*., acetate and glycerol, for the biosynthesis of both carbohydrates and lipids. Thus, by modulating physiological conditions at the end of the active growth phase, lipid accumulation could be increased. Further experiments are underway for optimizing growth conditions in mixotrophic and heterotrophic modes and nutrient limiting conditions to enhance lipid accumulation. The growth and lipid productivity of these cultures in outdoor cultivation system must also be assessed in future for large scale applications.

### Phylogenetic identification of selected microalgal isolates

We employed 18S rDNA analysis to phylogenetically identify our isolates. The partial sequences of 18S rDNA gene of TGA1, TGA2, TGA3 and PGA1 (919 bp, 633 bp, 748 bp & 835 bp) were submitted to GenBank under the accession numbers KM504962, KM504963, KM504964, and KM504965, respectively. These were further identified by phylogenetic analysis as *Acutodesmus dissociatus, Chlorella* sp*., Chlamydomonadales* sp*.* and *Hindakia tetrachotoma,* respectively (Additional file 2). The BLAST analysis of rDNA sequence of TGA1 exhibited 99% sequence similarity with *Acutodesmus* sp. TGA2 that was identified as *Chlorella* sp. had only 95% sequence similarity with *Chlorella* sp. available in the NCBI database. This may be due to a wide variation in the species composition of these unicellular and coccoid microalgae. Many reports have expressed the difficulty in identifying *Chlorella* as there are more than 100 *Chlorella* sp. described from the soil and fresh and marine water [23]. The BLAST analysis indicated 97% sequence similarity of TGA3 with *Chlamydomonadales* sp. available in the NCBI database. PGA1 isolate having unicellular and medium sized cells showed 91% sequence similarity with *Hindakia tetrachotoma*.

### Biomass and lipid productivity

The data on the comparison of biomass yield of the ten microalgal strains with the reference cultures at different stages of growth phases are given in Table 3. The microalgal cultures, TGA2, TGA3, TGA5, PBGA1, and PBGA2, exhibited maximum biomass yield during early log phase and strains such as TGA1, TGA4, PGA1, PGA2, and PBGA3 showed a maximum biomass yield after 12-days of cultivation, i.e., during the late growth phase. Irrespective of the cultures, the biomass yield decreased during the stationary phase. The biomass yield and cell dry biomass obtained in our samples are comparable with the values reported for the related species of green algae. The biomass yields recorded under this study were higher than those reported earlier [24]; 89 mg L^−1^ day^−1^ for *Chlorella* sp., 94 mg L^−1^ day^−1^ for *Scenedesmus* sp., 51 mg L^−1^d^−1^ for *Haematococcus* sp. and 78 mg L^−1^d^−1^ for *Nannochloropsis* sp. Owing to a rapid growth rate, *Chlamydomonadales* sp. isolated in this study might serve as a potential candidate for the bio oil production through HTL (hydrothermal liquefaction) even though it had lower lipid productivity than the reference strains.

**Table 3:** Biomass productivity of microalgal cultures at different stages of cultivation and lipid productivity of 16-day-old cultures. Data are given as means ±standard error, *n* = 3

Algal strain	Biomass productivity (mg L^−1^ d^−1^) at different days of cultivation				Lipid productivity (mg L^−1^ d^−1^)
	4	8	12	16	
*Acutodesmus dissociatus* TGA1	93.33 ± 11.59^bcde^	92.92 ± 9.43^abc^	119.44 ± 7.36^ab^	98.13 ± 2.73^ab^	22.27 ± 1.05^e^
*Chlorella* sp. TGA2	115.00 ± 16.4^abcd^	103.33 ± 6.23^abc^	113.89 ± 7.36^abc^	96.67 ± 3.28^abc^	27.12 ± 2.90^d^
*Chlamydomonadales* sp. TGA3	134.17 ± 16.87^ab^	81.67 ± 31.96^abc^	127.78 ± 5.56^a^	104.59 ± 4.34^a^	31.00 ± 0.11^c^
*Chlorella* sp. TGA4	59.17 ± 14.83^e^	54.58 ± 7.24^c^	72.21 ± 7.37^d^	62.29 ± 7.43^e^	10.81 ± 0.18^g^
*Chlamydomonadales* sp. TGA5	113.33 ± 17.66^abcd^	107.50 ± 8.76^ab^	108.33 ± 9.63^abc^	97.71 ± 3.51^ab^	23.11 ± 3.12^e^
*Hindakia tetrachotoma* PGA1	69.17 ± 9.40^de^	70.00 ± 7.23^bc^	88.89 ± 12.12^cd^	77.92 ± 4.88^d^	20.08 ± 0.28^ef^
*Chlorella* sp. PGA2	83.33 ± 10.94^cde^	65.83 ± 6.94^bc^	90.55 ± 9.70^bcd^	89.38 ± 3.09^bcd^	18.50 ± 1.07^f^
*Tolypothrix* sp. PBGA1	127.50 ± 15.90^abc^	72.92 ± 29.49^bc^	125.00 ± 4.82^a^	102.71 ± 4.72^a^	10.87 ± 2.80^g^
*Tolypothrix* sp. PBGA2	120.83 ± 14.55^abc^	95.83 ± 10.87^abc^	105.58 ± 7.36^abc^	86.25 ± 5.25^bcd^	7.85 ± 0.42^g^
*Oscillatoria* sp. PBGA3	90.83 ± 13.11^bcde^	92.50 ± 11.36^abc^	105.58 ± 7.36^abc^	84.17 ± 4.88^cd^	10.29 ± 1.15^g^
*Botryococcus* sp. REF1	151.67 ± 16.87^a^	126.08 ± 14.35^a^	127.77 ± 12.12^a^	105.42 ± 3.47^a^	43.31 ± 8.66^a^
*Neochloris oleoabundans* REF2	140.00 ± 15.90^ab^	112.50 ± 9.23^ab^	125.00 ± 12.75^a^	103.34 ± 4.71^a^	34.95 ± 5.31^b^

In a column, means followed by a common letter in superscript are not significantly different at 5%

Lipid productivity, which is a product of biomass and lipid content, is one of the most essential and pertinent features related to biofuel production. In this study, lipid productivity was recorded as milligrams of lipids produced per gram dry biomass per day. The lipid productivity of the tested strains after 16 days (Table 3) of cultivation ranged from 7.85 ± 0.42 mg L^−1^d^−1^ (PBGA2) to 31 ± 0.11 mg L^−1^d^−1^ (TGA3). TGA3 exhibited maximum lipid productivity owing to its high specific growth rate. Despite a significant yield of the biomass of PBGA1 (127.50 ± 15.90 mg L^−1^d^−1^) and shorter generation time, it was eliminated from further evaluation due to a lower lipid content (10.6%; Fig. 4).

A comparison of the performance of the microalgal isolates in the present study (Table 4) with existing reports indicates that *Acutodesmus dissociatus* (TGA1) is more productive due to its greater biomass productivity (119.44 ± 7.36 mg L^−1^d^−1^), faster specific growth rate (μ_exp_ - 0.23 day^−1^) and high lipid content (22.7%) over *Acutodesmus* sp. [5] and *Acutodesmus dimorphus* [25]. Similarly, *Chlorella* sp. (TGA2) exhibited maximum biomass yield (115.00 ± 16.4 mg L^−1^d^−1^) and lipid content (28%) and was twice of the specific growth rate (μ_exp_) of 0.25 d^−1^ of another *Chlorella* sp. [24]. The lipid content in *Chlamydomonadales* sp. (TGA3) was found to be higher than the *Chlamydomonadales* sp. strain DOE0101 [26]. The study also found *Hindakia tetrachotoma* (PGA1) with higher lipid content (25.70%), lipid productivity (20.08 ± 0.18 mg L^−1^d^−1^) and lower biomass (88.89 ± 12.12 mg L^−1^d ^−1^) over *Hindakia tetrachotoma* ME02 having biomass yield of 100 mg L^−1^d^−1^ [27]. Taken together, the findings suggest a possibility of obtaining potential oleaginous microalgal isolates with high biomass yield from natural resources by conducting more extensive exploration studies. Indeed, survey studies are still underway to obtain extremophilic microalgal isolates [28].

**Table 4:** Comparative analyses of biomass and lipid productivity, specific growth rate and lipid content of selected algal strains with available literature

Algal species	Biomass productivity	Specific growth rate	Lipid (%)	Lipid productivity	Reference
	(mg L^−1^ d ^−1^)	μ (day ^−1^)		(mg L ^−1^ d^−1^)	
*Acutodesmus dissociatus* TGA1	119.44 ± 7.36	0.23 ± 0.05	22.70 ± 0.46	22.27 ± 1.05	Current study
*Acutodesmus* sp.	–	–	14 ± 1.00	10 ± 8.00	Grama et al. [5]
*Acutodesmus dimorphus*	14.03	–	22.70	–	Chokshi et al. [25]
*Chlorella* sp. TGA2	115.00 ± 16.40	0.25 ± 0.03	28.00 ± 0.65	27.12 ± 2.90	Current study
*Chlorella* sp.	89.00	0.12–0.34	15.90	–	Andruleviciute et al. [24]
*Chlamydomonadales* sp. TGA3	34.17 ± 16.87	0.28 ± 0.03	29.70 ± 0.69	31.00 ± 0.11	Current study
*Chlamydomonadales* sp. DOE0101	–	–	25.00	–	Neofotis et al. [26]
*Hindakia tetrachotoma* PGA1	88.89 ± 12.12	0.18 ± 0.03	25.70 ± 0.59	20.08 ± 0.18	Current study
*Hindakia tetrachotoma* ME02	100 ± 0.50	4.6 ± 0.06	8.7 ± 1.90	10.00 ± 0.20	Onay et al. [27]
*Botryococcus* sp. REF1	151.67 ± 16.87	0.23 ± 0.08	41.00 ± 0.95	43.31 ± 8.66	Current study
*Neochloris oleoabundans* REF2	140.00 ± 15.90	0.24 ± 0.08	38.70 ± 0.89	34.95 ± 5.31	Current study

### Carbohydrate and protein profile of the isolates

For a cost effective and sustainable biodiesel production from microalgae, the utilization of residual algal biomass is also important. The defatted algal biomass consists primarily of carbohydrates and proteins, which can valorize biofuel based byproducts. The carbohydrate-enriched biomass of microalgae can serve as a substrate for methane generation or ethanol production. The protein rich microalgal biomass can be used as animal feed or a substrate for single cell protein production. Further, a high protein content of about 20% to 67% was reported by Renaud et al. [29] in the biomass of fresh water algae. The macromolecular composition of the microalgal isolates and the standard cultures is depicted in Fig. 4. Among the four selected cultures, PGA1 showed the maximum macromolecular (93.94%) composition. However, the total composition did not reach 100% in any of the cultures analyzed. This could be due to the presence of other components like pigments, nucleic acids, moisture, and ash content, which were not analyzed in this study. Of various microalgal strains, *Hindakia tetrachotoma* (PGA1) was recorded with the highest value for protein content (39.84%). A higher protein content was observed in algae grown in a 12:12 h light and dark regime [30]. While 16:8 h light and dark regime, adopted in the present study, might have enhanced photosynthetic accumulation of carbohydrate and substantially reduced the protein content. The best performing four microalgal cultures, TGA1, TGA2, TGA3, and PGA1, exhibited the carbohydrate content of 37.73 ± 1.96%, 42.50 ± 2.50%, 38.50 ± 2.26%, and 28.40 ± 2.26, respectively. Consequently, high carbohydrate content with less protein makes them good candidates for HTL technology, as mentioned above. “Lipid-extracted” algae may have additional benefits as the high protein rich feedstock [31].

### Growth under different temperature

**Fig. 5:**
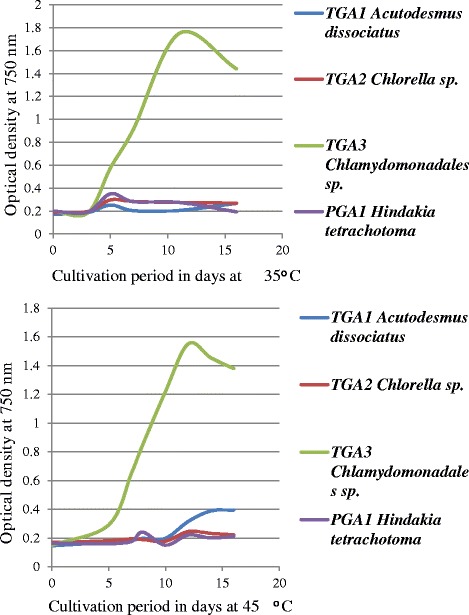
Growth pattern of four microalgal cultures grown at 35 ^ο^C and 45 ^ο^C

As India is a tropical country, native microalgal strains are capable of growth throughout the year. The TGA3 can grow normally even at 45 °C. However, an optimum growth of other three strains (TGA1, TGA2, and PGA1) was observed at 25 °C. Moreover, after 3–5 days of incubation at 35 °C and 45 °C, the cells of TGA1, TGA2 and PGA1 died and bleached. The growth pattern of TGA3 at 35 °C and 45 °C of incubation, respectively, is represented in Fig. 5. The culture reached maximum growth at day 11 and 12 when incubated at 35 °C and 45 °C, respectively. An insignificant variation in the growth of TGA3 was observed at these two temperatures. Due to the temperature differences, the evaporation and condensation of water vapor in growth flasks was observed when cultures were grown at 35 °C and 45 °C. These culture broths were examined microscopically to ensure that no contamination with bacteria occurred due to the condensation of water vapor. Most of the commercially available isolates are of temperate origin and are grown under controlled conditions [32]. Typically, the microalgal strains are susceptible to temperature stress and only a few degrees of increase in temperature results in bleaching and cell death [33]. The optimum temperature for growth of the microalgae ranges between 16 °C and 27 °C. The thermo-tolerant microalgal species like TGA3 that are adaptive to a wide temperature range confer an economic advantage by reducing the costs of input. However, the lipid content and fatty acid profile of the TGA3 grown at higher temperature should be investigated further to confirm its potential for open pond cultivation. Large scale outdoor cultivation systems are subjected to fluctuations in diurnal and seasonal temperatures. Thus native isolates are advantageous due to their inherent fitness to local conditions. Another important concern in open pond cultivation system is the risk of contamination. Taken together, the identification of microalgal strain that could overgrow predators and other inhabitants of the culture system is a very important consideration. Thermo tolerant strains could help sustain a monoculture in the cultivation system at an elevated temperature while reducing competition.

### Transesterification and GC-MS analyses of fatty acid profile of selected isolates

The ability of microalgae to produce biodiesel depends essentially on their fatty acid profile. Besides lipid productivity, the fatty acid composition is an important factor in screening algal strains for biodiesel production.

Biodiesel is mostly characterized by the presence of palmitic acid (C16:0), stearic acid (C18:0), oleic acid (C18:1), linoleic (C18:2) and linolenic acid (C18:3) methyl esters, and high levels of saturated and low levels of polyunsaturated fatty acids [33, 34]. Thus, the fatty acid chain length and degree of unsaturation are essential in determining fuel properties including kinetic viscosity (Vis), specific gravity (SG), cetane number (CN), cloud point (CP), iodine value (IV), and higher heating value (HHV) [35]. Importantly, cetane number, cloud point, and viscosity increases with an increase in chain length and decreases with increasing degree of unsaturation. On the other hand, the factors such as specific gravity, iodine value, and heating value increase with the increasing degree of unsaturation and decrease with the increasing chain length. Altogether, monounsaturated fatty acids are advantageous over polyunsaturated fatty acids (PUFA) for their desirable oxidative stability, cold flow, and combustion properties [36]. However, a certain degree of unsaturation could have a positive impact on the flow properties especially under the cold weather but have adverse effects on oxidative stability. Consequently, biodiesel with saturated fatty acids has optimum combustion properties, however, can cause cold flow problems [37].

The fatty acid profiles of 16-day-old cultures were identified by the GC-MS analysis. As shown in Table 5, the FAME profiles were found to be unique to specific strains. In all the best performing isolates, 39 compounds in total including fatty acids and related compounds were identified. The preponderance of fatty acid methyl esters, *viz*., C16:0, C18:1 and C18:3, was evident in more productive strains. Overall, palmitic acid (C16:0) was dominant in TGA1, TGA2, and PGA1, followed by oleic acid (C18:3) in fatty ester composition. As a common characteristic, the predominance of palmitic acid ranges between 20% and 27% in most algae and could reach as high as 49–50% [38]. However, TGA3 significantly contained a higher quantity of C18:3 (27.21%), which does not corroborate with an earlier report [39], which found that polyunsaturated C18 fatty acids are less prominent in algal oils than vegetable oils. In addition to C16 and C18 fatty acids, PGA1 is also found to have both lower (C3:0–11.20%) and higher chain (C19:1–10.43%) fatty acids. Despite a wide variation in the fatty acids profile, the percent saturation was slightly higher than the degree of unsaturation in TGA1 and TGA2. TGA3 exhibited a higher percentage of unsaturated fatty acids (42.17%); whereas, PGA1 had more saturated fatty acids (42.59%). Despite these variations, the fatty acid composition and the degree of unsaturation in the strains under study fall within the recommendations of biodiesel properties outlined by Song et al. and Hoekman et al. [34, 39].

**Table 5:** Fatty acid methyl ester profile of selected microalgal strains

Fatty Acid Methyl Esters	Fatty acid content (%)			
	TGA1	TGA2	TGA3	PGA1
C3:0 Propionic acid methyl ester	–	–	–	11.20 ± 3.26
C4:0 Butanoic acid methyl ester	0.95 ± 0.27	–	6.19 ± 1.78	2.88 ± 0.99
C8:0 Caprylic acid methyl ester	–	–	1.22 ± 0.38	–
C12:0 Lauric acid methyl ester	6.74 ± 1.94	0.63 ± 0.21	4.76 ± 1.48	6.20 ± 1.84
C14:0 Myristic acid methyl ester	2.35 ± 0.67	–	–	9.44 ± 2.74
C14:1 Myristoleic acid methyl ester	–	10.37 ± 3.05	11.15 ± 3.27	–
C16:0 Palmitic acid methyl ester	13.32 ± 3.85	14.06 ± 4.38	3.83 ± 1.23	12.50 ± 3.62
C16:1 Palmitoleic acid methyl ester	2.42 ± 0.70	–	–	–
C16:0 Palmitic alcohol	2.49 ± 0.72	11.42 ± 3.46	6.03 ± 1.74	–
C17:0 Heptanoic acid methyl ester	1.07 ± 0.34	–	–	
C18:0 Stearic acid methyl ester	–	0.22 ± 0.06	–	–
C18:1 Oleic acid methyl ester	11.75 ± 3.41	13.20 ± 4.58	2.64 ± 0.76	–
C18:3 Linolenic acid methyl ester	11.70 ± 3.51	6.63 ± 1.91	27.21 ± 8.10	13.45 ± 3.91
C19:0 Nonadecanoic acid methyl ester	5.07 ± 1.58			
C19:1 Nonadecenoic acid methyl ester	–	–	–	10.43 ± 3.01
C20:1-Eicosenoic acid methyl ester	–	–	1.03 ± 0.40	–
C22:0 Behenic acid methyl ester	3.02 ± 0.95	–	–	–
C22:4 Docosatetraenoic acid methyl ester	–	1.36 ± 0.41	–	–
Diisooctyl phthalate (Volatile oil)	24.71 ± 3.20	14.06 ± 2.10	29.89 ± 4.89	–
Dasycarpidan–1-methanol, acetate (ester) (Volatile oil)	2.57 ± 0.78	5.29 ± 0.54	4.39 ± 0.88	11.44 ± 4.30
Phthalic acid, di(2-propylpentyl) ester	–	11.20 ± 3.21	–	–
Others	11.33 ± 3.78	10.20 ± 2.67	1.12 ± 0.41	21.01 ± 6.30
Total	99.68	99.14	99.67	99.13
Saturated fatty acids (%)	35.12	26.56	22.10	42.59
Unsaturated fatty acids (%)	25.95	31.83	42.17	24.09

Data shown are mean value of three replicates ±standard error

TGA1- *Acutodesmus dissociatus;* TGA2-*Chlorella* sp.; TGA3-*Chlamydomonadales* sp.; PGA1- *Hindakia tetrachotoma*

Besides, certain additional types of fatty acids of medicinal and industrial importance were also observed in the four isolates. TGA1 had a volatile oil, di-iso-octyl phthalate (24.71%), with plasticizing capacity and a sterol (9–10-seccholesta 5, 7, 10, 19 triene-1-3-diol, 2–5 trimethyl silyl oxy-(3, 5, Z-7-E)) with medicinal properties. Lower chain fatty acid derivatives were also found to present in the fatty acid profile (phthalic acid, di (2-propylpentyl) ester, 11.20%) of TGA2. It showed the presence a hydrocarbon of 17-pentatriacontene (C_35_ H_72_).

TGA3 exhibited the maximum concentration of diisooctyl phthalate as 29.89%. The fatty acids such as butanoic acid (C4:0, 6.19%), caprylic acid (C8:0, 1.22%) dodecanoic acid (C12:0, 4.76%) and myristoleic acid (11.15%) were also found in TGA3. The higher levels of palmitic alcohol were also noticed. TGA3 contained higher chain fatty acid such as cis-11-eicosenoic acid and the traces of long chain hydrocarbon of tetrapentacontane (C_54_ H_110_).

*Hindakia tetrachotoma* (PGA1) exhibited a 16.18% content of 9-(2′, 2′-dimethyl propanoyl hydrazono)-3, 6-dichloro-2, 7-bis-[2-(diethyl amino)-ethoxy] fluorene. PGA1 showed maximum value for dasycarpidan-1-methanol acetate of 11.44%. PGA1 comprised of many saturated fatty acids, *viz*., propionic acid, butanoic acid, lauric acid, and myristic acid. In addition, PGA1 exhibited a unique glycolipid compound, D-mannitol-1-decyl-sulfanul and a vitamin D supplement-androstane-11, 17-dione,3-[(trimethylsilyl)oxy]-,17-[O-(phenylmethyl)oxime] in trace quantity. The results of FAME analysis thus confirmed that algal fatty acids are of diverse nature including with shorter (C3 − C14) and longer chain (C19, C20, and C22) fatty acids [30]. Wu et al. [40] suggested that lipids with high unsaturated fatty acid, especially oleic acid are better feedstock for high-quality biodiesel production. The presence of significant amounts of C14:0, C14:1, C16:0, C18:1 and C18:3 in our study indicates that biodiesel from the selected algae would achieve high cetane number to meet the biofuel standards of ASTM D6751 in the US and EN 14214 in Europe [41]. Hence, the selected four strains are potential candidates for biodiesel production meeting the global standards of fuel properties.

## Conclusions

The present investigation is a first systematic survey on the microalgal diversity that illustrates the isolation, characterization and evaluation of oleaginous microalgal species *Acutodesmus dissociatus* (TGA1), *Chlorella* sp. (TGA2), *Chlamydomonadales* sp. (TGA3) and *Hindakia tetrachotoma* (PGA1) from the shola forests of Western Ghats of the NBR, India. The most productively promising oleaginous microalgae were obtained from Top Slip (TGA1, TGA2, and TGA3) and Parambikulam (PGA1) of NBR with maximum species richness. Specifically, the present study successfully isolated and characterized ten potent oleaginous microalgal strains and found three major fresh water green algae and a novel oleaginous green alga *Hindakia tetrachotoma*, as promising feed stocks for biodiesel production. The *Chlamydomonadales* sp. (TGA3) was found to be significantly thermo-tolerant and can be considered as promising feedstock for biodiesel production. Furthermore, FAME analysis of the four isolates suggests the use of these cultures as important alternative sources for industrially relevant oil based compounds**.** In particular, the presence of a remarkable accumulation of carbohydrate and protein in the isolated strains offers further scope for valorization**.** Further comprehensive studies on these isolates are needed to optimize growth under different stress conditions and to assess the economic feasibility of mass production for sustainable biofuel production processes with multiple benefits.

## Methods

### Sample collection and isolation of microalgal cultures

Microalgae rich soil (18), water (12), and bark with lichens (19) were collected from eighteen locations of the NBR, India during March 2013 (temperature 22 to 37 °C and pH 4.5 to 8.5).

The isolation of algae from natural samples requires the use of multiple media in order to ensure the recovery of all major algal species present in the samples. Thus, microalgal samples were further enriched by transferring 1 g soil sample/1 mL water sample/bark sample with lichens to glass test tubes containing 10 mL broth of either BG11 (with and without nitrogen source) to isolate both green algae and blue green algae or 10 mL Chu13 modified media for isolation of green algae. An antibiotics mixture at a concentration of 10 ppm of streptomycin sulfate, 5 ppm chloramphenicol, and 1 ppm ketoconazole [42, 43] was also supplemented to both media to arrest the growth of bacterial and fungal contaminants without inhibiting the growth of microalgae. The tubes were incubated at 24 ± 1 °C under continuous illumination (35 μmol photons m^−2^ s^−1^) by fluorescent lamps with the light and dark cycle of 16:8 h for 30–40 days. The samples under enrichment were observed regularly under a MAGNUS MLX (Olympus) compound microscope. The enriched samples were taken periodically and wet mounts were examined microscopically for the presence of different algal forms like unicellular, colonial, filamentous, and heterocystous algae. Morphologically distinct genera were recorded and the data were used for algal diversity analysis. The presence and dominance of particular type of algae such as diatoms, chlorophytes, and cyanophytes during enrichment period were observed and scored. Using this qualitative data, the microalgal succession was analyzed. Further, these microalgal cultures were purified by serial dilution and plating technique. Microscopic observation of purified cultures confirmed the presence of unialgal cultures. These unialgal cultures were used for further identification by morphological studies. Additional microalgal strains such as *Botryococcus* sp. and *Neochloris oleoabundans* were obtained from culture collection center of TNAU, Coimbatore and University of Agricultural Sciences, Dharwad, India and included as reference.

### Identification, characterization, and growth of the cultures

The isolated microalgae were identified by analyzing the standard morphological features under MAGNUS MLX (Olympus) compound microscope using the keys given in standard monographs [17, 18]. The identified cultures were grown in three different media, viz., BG11, modified Bold’s Basal Medium (BBM), and Bristol medium supplemented with antibiotics, at 24 ± 1 °C, under uninterrupted light (35 μmol photons m^−2^ s^−1^) by fluorescent tubes (Philips) set on a 16:8 h light and dark cycle with a timer for 30–40 days. The best medium was chosen for further studies.

### Growth of the most productive microalgal cultures under different temperature

In order to select microalgal strains for open raceway cultivation, the isolates were grown at different temperatures 25 °C, 35 °C, and 45 °C and the growth pattern was monitored spectrophotometrically by measuring the optical density at 750 nm.

### Microalgal diversity

Microalgal diversity was calculated as described previously by Magurran [44]. Microalgal diversity based on species richness, evenness, and dominance was measured using Shannon-Wiener index (H′), Shannon’s equitability index (E_H_), and Simpson index (D), respectively. Species richness was measured using Shannon diversity index [45]. This presents the Shannon-Wiener (also known as Weaver) diversity index for each sample and is defined as

$$ {H}^{'}\kern0.5em =\kern0.5em \Sigma \kern0.5em {p}_i\kern0.5em \ln\;{p}_i $$

where *pi* = *n*_*i*_*/N*; *n*_*i*_ = the abundance of the i^th^ species in the sample; *N* = the total abundance. The equitability or evenness was calculated using Shannon’s Equitability (EH) index, which refers to the pattern of distribution of the individuals among the species [46]. The equitability index compares the observed Shannon-Wiener index to that of the distribution of individuals between the observed species which would maximize diversity. Equitability assumes a value between 0 and 1 with one being complete evenness. Therefore, the index is equated as:

$$ {E}_H=\kern0.5em {H}^{'}/{H}_{max}={H}^{'}/ lnS $$

where H^′^ is the observed Shannon-Wiener index and S is the total number of species in the habitat.

Species dominance was assessed using Simpson’s index [47], which describes the probability that two individuals drawn at random from a population belong to the same species.

$$ \mathrm{Simpso}{\mathrm{n}}^{\prime}\mathrm{s}\ \mathrm{Index}\ \left(\mathrm{D}\right)\kern0.5em =\kern0.5em \Sigma \left[{n}_i\left({n}_i\hbox{--} 1\right)\left]/\right[N\left(N\hbox{--} 1\right)\right] $$

where *n*_*i*_ = the number of individuals in the i^th^ species and *N* = the total individuals in the sample.

### Screening microalgae for biomass production

Microalgal cultures were screened for biomass yield considering their dry cell weight, specific growth rate, generation time, and chlorophyll content.

The growth profile of the isolates cultured in batch mode was monitored spectrophotometrically and gravimetrically. The biomass yield was measured using dry weight, and for the quantitative analysis and the calculation of generation time and specific growth rate, the optical densities were measured at 750 nm.

For the determination of the growth characteristics, microalgal cultures were first grown in 500 mL glass Erlenmeyer flasks containing 200 mL of modified Bold’s Basal Medium (BBM) by inoculating with 2% starter culture. The flasks were incubated at 24 ± 1 °C under continuous illumination with 16:8 h light and dark cycle for 16 days. The initial cell concentration was kept constant (OD _750_ = 0.2–0.3) by diluting with the medium. Of the sample, 1 mL was harvested to measure the optical density at 750 nm. The microalgal dry weight was determined in triplicate once in every 4 days up to 16 days. For the dry weight measurement, approximately 10 mL microalgal suspension was centrifuged at 4000 rpm for 10 min. The algal pellet was washed twice with distilled water to remove the salts and then the harvested biomass was oven dried at 105 °C until it reached a constant weight and the dry biomass was expressed as g L^−1^. The biomass productivity (*P*_Biomass_), which was analyzed at different time points during growth, was calculated using the following equation and represented as mg L^−1^ d^−1^.

Biomass productivity (mg L^−1^ d^−1^), = (W_2_-W_1_)/(t_2_-t_1_) where,) W_2_ and W_1_ are the concentration of the dry biomass (g L^−1^) at time t_2_ and t_1,_ respectively.

The specific growth rate (μ) for the period between an initial (t_1_) and final (t_2_) day of growth was calculated from the corresponding algal biomass, Wo, and W_f_ as follows [48].

$$ \upmu\ \left({\mathrm{day}}^{\hbox{--} 1}\right)=\ln\ \left({\mathrm{W}}_{\mathrm{f}\hbox{--} }{\mathrm{W}}_{\mathrm{o}}\right)/{\mathrm{t}}_2\hbox{-} {\mathrm{t}}_1 $$

The generation time of the isolates was calculated using the optical density measured during log phase growth [49].

G *= 1/R,* where G is the generation time and ‘R’ is the specific growth rate of logarithmic cells.

The chlorophyll content of 16-day-old microalgal cultures was estimated with modification to the methanol extraction method [50]. About 10 mL of algal culture was centrifuged at 4000 rpm for 10 min and the resultant pellet was treated with a known volume of methanol and kept in water bath for 30 min at 60 °C. The absorbance of the pooled extracts was measured at 645 nm and 665 nm (Varian; Cary50) and chlorophyll content (a and b) was calculated using standard equations.

### Screening microalgae by Nile red staining

Nile Red stain (1 mg dissolved in 10 mL acetone) was used to stain the microalgal cultures. About 0.5 mL of 12-day- old microalgal cultures was centrifuged at 5000 rpm for 10 min. The collected biomass was dissolved in normal saline and centrifuged at 5000 rpm for 10 min and the pellet was collected. All experiments were repeated four times. The collected pellet was dissolved in a small quantity of normal saline and then Nile Red stock solution was added at the ratio of 100:1 (*v*/v) and incubated for 20 min. The mixture was then observed under Fluorescent Microscope (NIKON; Eclipse H600L) using excitation filter of 450–490 nm and 570 nm emission wavelengths.

### Screening microalgal cultures for macromolecular production

All the ten microalgal cultures including reference strains were grown in 500 mL glass conical flasks containing 200 mL modified BBM broth and incubated at 24 ± 1 °C with 16:8 h light and dark cycle. Microalgal cultures were harvested at day 16 and analyzed for carbohydrate, protein and lipid contents by following standard protocols. The carbohydrate content of 16-day-old cultures was determined by the modified phenol-sulfuric acid method after acid hydrolysis of the sample using glucose as a standard [51]. The protein content was determined using Lowry’s method [52] after alkaline hydrolysis of the sample and using Bovine Serum Albumin (BSA) as a standard.

The total lipid content of the microalgal cultures was determined following the procedure described by Folch et al. [53]. The powdered dry algal biomass of 100 mg was macerated with 5 mL of a mixture containing methanol and chloroform (2:1) and centrifuged at 4000 rpm for 10 min. The organic layer containing lipid was separated, dried, and the lipid content was assessed gravimetrically.

$$ \mathrm{Lipid}\  \mathrm{productivity}=\mathrm{Biomass}\  \mathrm{productivity}\ \left(\mathrm{mg}\ {\mathrm{L}}^{\hbox{--} 1}{\mathrm{d}}^{\hbox{--} 1}\right)\times \mathrm{Lipid}\  \mathrm{content}\ \left(\%\right) $$

### Identification of the most productive microalgal cultures by 18S rDNA analysis

The genomic DNA from the four microalgal cultures was isolated using hexadecyl-trimethyl-ammonium bromide (CTAB) method with minor modifications [54]. The quantity and quality of the DNA were determined using a Nano drop Fluorometer at OD_260/280nm_ for DNA. The 18S rDNA gene of each isolate was amplified using the universal primers; forward primer CV1 (5’-TACCTGGTTGATCCTGCCAGTAG–3′) and reverse primer CV2 (5′- CCAATCCCTAGT CGGCATCGT–3′). For PCR amplification, a reaction mixture (20 μL), containing 50 ng DNA template (2 μL), 1X Taq buffer (2 μL), 0.2 mM of each dNTP mixture (2 μL), 1 μM of forward primer (0.5 μL), 1 μM of reverse primer (0.5 μL), 1.5 mM MgCl_2_ (1 μL) and 2 Units of Taq DNA polymerase (0.5 μL) (Bangalore Genei, India), and distilled water (11.50 μL), was prepared. PCR amplification was carried out in a Thermocycler (Quanta Bio, USA) with the initial denaturation temperature of 95 °C for 5 min followed by 35 cycles at 95 °C for 30 s, annealing at 45 °C for 30 s, extension at 72 °C for 2 min with a final extension at 72 °C for 10 min. Sequence analysis was then performed on PCR products using BigDye® Terminator kit on ABI 3730XL automatic DNA sequencer (Applied Biosystems). Bioedit v.7.2.0 was used to assemble the 18S rDNA gene sequences. A phylogenetic tree was constructed using the Neighbor-Joining (NJ) method [55] as implemented in the program MEGA v.6.0 with bootstrapping at 1000 replicates. Microalgae included in the phylogenetic tree were chosen from the NCBI BLAST search.

### Transesterification and fatty acid profiling

#### Transesterification

Fatty acid methyl ester (FAME) was prepared by single step extraction and transesterification method [56]. Approximately 100 mg dry biomass of 16-day-old culture was dissolved in 10 mL mixture containing methanol, concentrated sulfuric acid, and chloroform in the ratio of 4.25: 0.75:5.00. The transesterification was carried out at 90 °C for 5 h in a water bath. On completion of the reaction, the mixture was centrifuged at 5000 rpm for 15 min and the FAME containing chloroform layer was separated using anhydrous sodium sulfate.

### Fatty acid profiling – GC-MS analysis of FAME

The GC-MS analysis was performed using the Trace GC-Ultra Thermo scientific instrument. The oven temperature was set at 80 °C, held for 5 min and then raised to 300 °C at a rate of 5 °C per min and held at 300 °C for 5 min. The injector temperature was set at 270 °C and the carrier gas (Helium) was controlled at 1 mL/min. The compounds were identified in the NIST Mass Spectral Database and quantified by the area normalization method.

### Statistical analysis

All statistical analyses were performed using SPSS Statistics 16.0 software. All data presented in tables and figures are expressed as the mean ± standard error. The growth rate, biomass and lipid content data for microalgal strains were analyzed using a one-way analysis of variance (ANOVA) and Duncan’s post hoc analysis. Differences were considered significant at *p* < 0.05.

## Additional files

Additional file 1: **Table S1.** Microalgal species composition of different samples. (DOCX 18 kb)

Additional file 2: xml file of phylogenetic data. (XML 50 kb)

## Abbreviations

GC-MS: Gas Chromatography Mass Spectroscopy
HTL: Hydrothermal Liquefaction
NBR: Nilgiri Biosphere Reserve
PUFA: Polyunsaturated Fatty Acid
SFA: Saturated Fatty Acid
TAG: Triacylglycerol
UFA: Unsaturated Fatty Acid
