# Supplementary material for: Growth and metabolic characteristics of oleaginous microalgal isolates from Nilgiri biosphere Reserve of India
Source: BMC Microbiol. 2018 Jan 3;18:1. doi: 10.1186/s12866-017-1144-x (PMC5809961; doi:10.1186/s12866-017-1144-x)
Supplement: Supplementary file 1 — Table S1. Microalgal species composition of different samples. (DOCX 18 kb) [file 12866_2017_1144_MOESM1_ESM.docx]

**Table S1** Microalgal species composition of different samples*

| Location | Type of sample | Cyanophyceae | | | Chlorophyceae | | Bacillariophyceae |
| --- | --- | --- | --- | --- | --- | --- | --- |
|  |  | Filamentous and heterocystous | Filamentous and Non heterocystous | Unicellular | Filamentous | Unicellular |  |
| Maize field of eastern block, TNAU | Soil | *Nostoc, Anabaena,*  *Tolypothrix* | *Oscillatoria* | *Microcystis Chroococcus, Gleocapsa,* | - | *Chlorella, Scenedesmus, Chlorococcum,*  *Dactylococcus* | Diatom |
| Wet land, TNAU | Water | *Tolypothrix* | *Oscillatoria Spirulina* | *Aphanocapsa Microcystis Gleocapsa Chroococcus Aphanothece Synechocystis,*  *Synecococcus,* | *Spirogyra* | *Chlorella, Chlorococcum Scenedesmus Chlamydomonad*  *Dactylococcus,* | *Navicula* |
| SethumadaiNilgiri Biosphere Reserve | Soil | *Tolypothrix Nostoc Calothrix*  *Anabaena* | *Oscillatoria Lyngbya* | *Gleocapsa* | - | - | - |
| Top Slip, Nilgiri Biosphere Reserve | Soil | *Tolypothrix Nostoc Calothrix* | *Oscillatoria Lyngbya* | *Gleocapsa Synecoccocus Chroococcus* | - | *Chlorella Chlorococcum Chlamydomonad* | Diatom |
| Top Slip, Nilgiri Biosphere Reserve | Water | *Tolypothrix* | - | *Gleocapsa Chroococcus Synecococcus* | - | *Chlamydomonad*  *Dactylococcus Chlorella Scenedesmus Chlorococcum* | *Navicula* |
| Parambikulam, Nilgiri Biosphere Reserve | Soil | *Cylindrospermum Tolypothrix* | *Oscillatoria, Lyngbya* | *Chroococcus Synecococcus* | - | *Scenedesmus*  *Chlamydomonad, Dactylococcus* | Diatom |
| Parambikulam, Nilgiri Biosphere Reserve | Water | *Tolypothrix* | *Oscillatoria Lyngbya*, | *Microcystis Chroococcus* | *-* | *Chlorella Chlorococcum Hindakia Scenedesmus Botryococcus*  *Dactylococcus* | *Navicula*  Unknown |
| Kamarajar sagar dam, Nilgiri Biosphere Reserve | Water | - | *Oscillatoria*, | *Microcystis* | - | *Scenedesmus*  *Dactylococcus* | Diatom |
| Kamarajar sagar dam, Nilgiri Biosphere Reserve | Soil | - | - | *Aphanocapsa Synecococcus* | - | *Chlorococcum*. | - |
| Kamarajar sagar dam, Nilgiri Biosphere Reserve | Bark | - | - | - | - | *Trentephila* | - |
| Shola forest, Nilgiri Biosphere Reserve | Soil | *Anabaena* | *Lyngbya* | *Merismopodia Chroococcus, Aphanocapsa* | - | *Chlorella* | - |
| Mel Kuntha, Nilgiri Biosphere Reserve | Soil | - | *Oscillatoria* | - | - | *Chlorella* | - |
| Rose garden, Ooty | Soil | *Nostoc, Anabaena* | - | - | - | - | - |
| HPF,Ooty | Soil | *Gleotrichia* | *Lyngbya* | *Synecococcus* | - | *Chlorella Scenedesmus*  *Dactylococcus* | - |
| Pykara –Nilgiri Biosphere Reserve | Water | - | - | *Microcystis* | - | *Scenedesmus*  *Dactylococcus* | - |
| Pykara –Nilgiri Biosphere Reserve | Soil | - | - | *Chroococcus* *Gleocapsa* | - | - | - |
| Total | | 19 | 15 | 31 | 1 | 37 | 8 |

*Morphologically identified utilizing the keys of [4][5].
